# High seroprevalence of *Rickettsia* spp. and molecular detection of *Rickettsia amblyommatis* in human-biting ticks from the eastern Amazon, Brazil

**DOI:** 10.1186/s13071-025-06944-3

**Published:** 2025-08-04

**Authors:** Mayra F. F. R. Ferreira, Lina de Campos Binder, Rafael M. S. Nogueira, Ruth M. M. e Silva, Carlos C. M. Ramos, Leonardo T. Dall’Agnol, Thiago Fernandes Martins, Raphaela B. M. Bittencourt, Felipe da Silva Krawczak, Livio Martins Costa-Junior, Marcelo Bahia Labruna, Mayara I. S. Lima, Hermes Ribeiro Luz

**Affiliations:** 1 Post‑Graduation Program in Health and Environment, Federal University of Maranhão, São Luís, MA Brazil; 2 Department of Preventive Veterinary Medicine and Animal Health, Faculty of Veterinary Medicine and Animal Science, University of São Paulo, São Paulo, Brazil; 3 Post‑Graduation Program in Northeast Biotechnology Network (RENORBIO), Federal University of Maranhão, São Luís, MA Brazil; 4 Post‑Graduation Program in Biodiversity and Conservation, Federal University of Maranhão, São Luís, MA Brazil; 5 Laboratory of Parasitic Diseases (LADOPAR), Department of Preventive Veterinary Medicine, School of Veterinary and Animal Science, Federal University of Goiás (UFG), Goiânia, Brazil; 6 Post‑Graduation Program in Animal Health Defense, State University of Maranhão, Maranhão, Brazil; 7 Post‑Graduation Program in Health Sciences, Center of Biological and Health Sciences, Federal University of Maranhão, São Luís, MA Brazil

## Abstract

**Background:**

In Brazil, spotted fever (SF) is caused by the bacteria *Rickettsia parkeri* and *Rickettsia rickettsii*. Seroepidemiological data on *Rickettsia* spp. in humans are rare in Brazil and nonexistent in the Amazon biome. We sought to quantify antibodies reactive to *Rickettsia* spp. in serum samples collected from humans in the Amazonia biome, and to detect *Rickettsia* spp. in ticks parasitizing these hosts.

**Methods:**

Human blood samples were collected from three different locations within the eastern Amazon in Maranhão State, northeastern Brazil between 2010 and 2018. Sera generated from those samples were tested for the presence of antibodies reactive to *Rickettsia* by immunofluorescence assay (IFA) using crude antigens from five *Rickettsia* isolates from Brazil: *R. rickettsii* strain Taiaçu, *R. amblyommatis* strain Ac37, *Rickettsia rhipicephali* strain HJ5, *R. parkeri* strain At24, and *Rickettsia bellii* strain Mogi. Between 2020 and 2025, ticks were manually collected while attached to the skin of humans in seven municipalities in Maranhão State. Adult ticks were randomly selected and individually processed for DNA extraction and examined using a real-time PCR (qPCR) assay targeting a fragment of the rickettsial *gltA* gene. The qPCR-positive samples were subsequently examined by conventional PCR (cPCR) targeting the *ompA* gene of SFG rickettsiae. The cPCR amplicons were purified and sequenced bidirectionally using the amplification primers. The resulting sequences were compared with those in GenBank using BLASTn to identify related *Rickettsia* spp.

**Results:**

A total of 341 human serum samples were analyzed, and 145 (42.5%) were recorded as reactive with at least one species of *Rickettsia*. Among the reactive samples, 68 (47%) were from Imperatriz, 45 (31%) from Açailândia, and 32 (22%) from the municipality of São Luís. *Rickettsia rhipicephali* was recorded as the possible antigen involved in a homologous reaction (PAIHR) in one individual, *R. amblyommatis* in five, and *R. bellii* in three. A total of 187 ticks were collected parasitizing humans in the Amazon biome. Molecular analyses revealed that *Rickettsia* DNA was present in 44.4% (4/9) of *A. cajennense* s.s. from Açailândia, 52.4% (22/42) from Centro Novo do Maranhão, and 0% (0/8) from Imperatriz. Among the adults of *A. coelebs* from Centro Novo do Maranhão, 36.8% (7/19) tested positive. In contrast, all adults of *A. oblongoguttatum* from the same location tested negative (0/20).

**Conclusions:**

This is the first study to detect anti-*Rickettsia* antibodies in humans and to identify *R. amblyommatis* in ticks parasitizing humans in the Amazon biome. The detection of *R. amblyommatis* in human-biting ticks, together with concurrent seropositivity in human sera from the same region, supports the hypothesis that this agent is actively circulating in the Amazon biome and may be responsible for undiagnosed cases of nonlethal spotted fever in the area.

**Graphical abstract:**

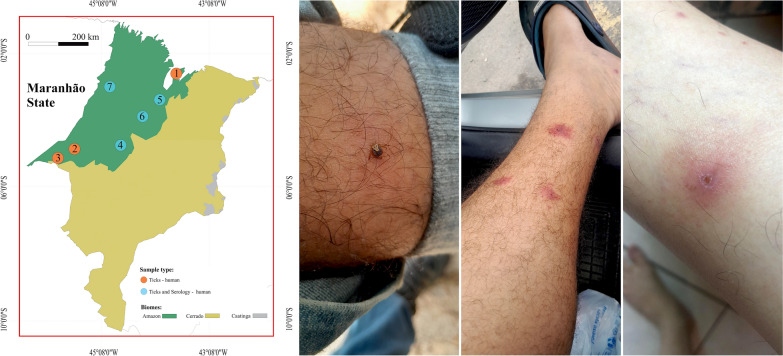

## Background

Spotted fever (SF) is a zoonosis caused by spotted fever group (SFG) rickettsiae and transmitted to humans by arthropod vectors, mainly ticks [1, 2]. Currently, ~19 tick-borne SFG rickettsiae have been associated with human diseases globally, with emphasis on *Rickettsia parkeri* (nonlethal) and *Rickettsia rickettsii* (lethal), the main agents of human SF in Brazil [2–5]. Both agents are responsible for SF in the southeast and south regions of Brazil, while *R. parkeri* has also caused SF in the northeast region [2, 5, 6].

The northeast region has seen an increase in SF cases over the last decade [6, 7]. At the same time, several knowledge gaps have emerged—in this context, more than half of the confirmed cases in the northeast region do not have a clearly identified causative agent, being reported only as *Rickettsia* sp., and the vector is undetermined. To date, the *Rickettsia* species has been registered in only two human cases, both caused by *R. parkeri* [5, 8].

Although a presumably fatal case of SF was reported in 2018 in the northeast region [9], the diagnostic methods used were insufficient to confirm it as SF, as the polymerase chain reaction (PCR) assay could not differentiate between rickettsial groups (e.g., SFG or typhus group), and no reliable DNA sequences could be obtained from the PCR products. Additional spotted fever group (SFG) *Rickettsia* species may be responsible for causing nonlethal spotted fever (SF) in humans in northeastern Brazil, including the Amazon biome. However, limited knowledge about these agents hinders accurate diagnosis, which directly impacts treatment and hampers the epidemiological understanding of SF. It may also interfere with the treatment of other diseases with similar symptoms, e.g., arbovirus associated febrile illnesses including dengue and chikungunya.

Although Maranhão State is considered a component of the northeast region, a large portion of its territory is covered by the eastern Amazon biome (~40%), which transitions into the Cerrado biome (~55%) [10]. The eastern Amazon also spans other Brazilian states (Pará, Tocantins, Amapá, and Mato Grosso) and hosts a high diversity of birds and mammals that serve as hosts for numerous tick species. Several of these ticks have been recorded parasitizing humans in Brazil, including *Amblyomma humerale*, *Amblyomma geayi*, *Amblyomma calcaratum*, *Amblyomma coelebs*, *Amblyomma oblongoguttatum*, *Amblyomma ovale*, *Amblyomma cajennense* sensu stricto (s.s.), and occasionally *Amblyomma sculptum* in the Amazon–Cerrado transition zone [11–14].

In the Amazon biome, SFG *Rickettsia* agents have been detected in numerous tick species, as well as in domestic and wild animals [14–21]. Yet, the possibility of SF in humans in this biome has been largely neglected. It is worth noting that *A. ovale* is the vector of *R. parkeri*, and *A. sculptum* is the vector of *R. rickettsii*. In the Amazon biome, *A. coelebs*, *A. oblongoguttatum*, and *A. cajennense* s.s. represent potential vectors of *Rickettsia amblyommatis*, an agent that may be responsible for SF cases in the USA [22].

In this context, we can infer that the eastern Amazon (Maranhão State) represents a silent region for human SF cases. Therefore, its inclusion in epidemiological surveillance studies should be considered essential, as highlighted by Oliveira et al. [23].

The indirect immunofluorescence assay (IFA) represents the most suitable method for seroepidemiological studies of SF and is considered the gold standard for the serological diagnosis of rickettsial infections in humans [24, 25]. This technique is based on the detection of immunoglobulin (Ig)M or IgG class antibodies using species-specific rickettsial antigens. In patients infected with *Rickettsia* spp., IgG levels can remain elevated for months or even years, underscoring the importance of IFA in seroepidemiological studies of SF. Serological surveys of *Rickettsia* spp. in humans are scarce in silent regions for SF in Brazil and are nonexistent in the Amazon biome. Furthermore, little is known in relation to the associations between ticks, *Rickettsia* spp., and humans in this biome. Thus, the present study aimed to detect anti-*Rickettsia* spp. antibodies in the blood collected from humans in different municipalities of the eastern Amazon portion of Maranhão State, and to identify *Rickettsia* spp. in ticks parasitizing humans in the same region.

## Methods

**Fig. 1:**
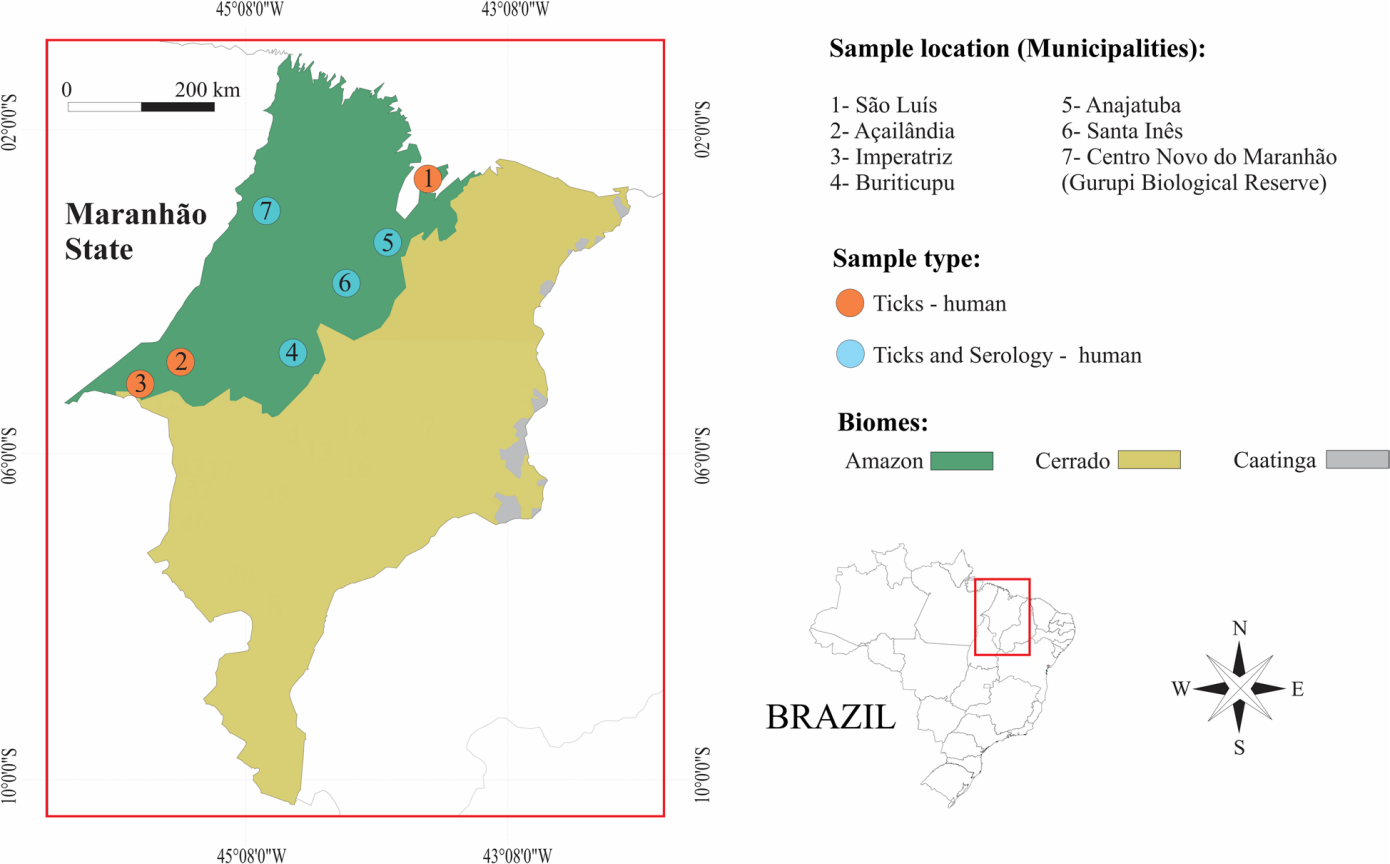
Map indicating locations where blood and ticks were collected from humans, Amazon biome, Maranhão State, Brazil

Human blood samples were collected, between 2010 and 2018, from three different locations within the eastern Amazon in Maranhão State, northeastern Brazil (Fig. 1): São Luís (2° 31′ 48″; 44° 18′ 10″), Imperatriz (5° 31′ 33″; 47° 28′ 33″), and Açailândia (4° 56′ 49″; 47° 30′ 18″). Patient ages were recorded only in the cities of São Luís and Imperatriz, ranging from 2 to 67 years. Blood samples were obtained through the project “development of molecular and immunological platforms for the diagnosis and monitoring of leprosy,” conducted by the Federal University of Maranhão (protocol no. 24680319.1.0000.5087).

The serum samples were sent to the Parasitic Diseases Laboratory at the University of São Paulo, where they were centrifuged (1500 × *g* for 10 min), aliquoted into microtubes, and stored at −20 °C for subsequent serological testing. Human sera were examined via an indirect immunofluorescence assay (IFA) protocol using crude antigens from five Brazilian *Rickettsia* isolates: *R. rickettsii* strain Taiaçu, *R. amblyommatis* strain Ac37, *Rickettsia rhipicephali* strain HJ5, *R. parkeri* strain At24, and *Rickettsia bellii* strain Mogi. All species have been reported in *Amblyomma* ticks known to parasitize humans in Brazil [26, 27].

Human sera were serially diluted in twofold increments with phosphate-buffered saline (PBS), starting from a 1:64 dilution. In total, 10 µL of the diluted serum were added to each well of the antigen slides. The slides were incubated at 37 °C for 30 min in a humidity chamber, then rinsed once and washed twice for 15 min with PBS. After incubation, the slides were stained with fluorescein isothiocyanate and washed again as described above. They were then mounted with buffered glycerine under coverslips and examined by fluorescent microscopy employing an Olympus model BX60 apparatus at 400× magnification.

The endpoint titer of reactivity towards each of the five *Rickettsia* antigens was determined. Reactive sera were tested in two or three replicates before the endpoint titer was established. Each slide included a serum previously shown to be nonreactive (negative control) and a serum known to be reactive (positive control), both tested at a dilution of 1:64. The positive control serum was previously shown to react with *R. amblyommatis*, *R. rhipicephali*, *R. rickettsii*, *R. parkeri*, and *R. bellii*, with endpoint titers ranging from 128 to 1024.

Any samples of human serum that showed an endpoint titer for a given *Rickettsia* species at least four times higher than the titers recorded towards the four other *Rickettsia* species was considered homologous to that species or to a very closely related genotype.

Between 2020 and 2025, ticks were manually collected from the skin of humans (researchers and rural workers) in seven municipalities of Maranhão State: São Luís, Açailândia, Anajatuba, Santa Inês, Buriticupu, Imperatriz, and Centro Novo do Maranhão (Fig. 1). In Centro Novo do Maranhão, ticks were collected at the Gurupi Biological Reserve, a large natural area within the Amazon biome.

Ticks were identified using the criteria recommended by Dantas-Torres et al. [28]. In addition, ticks belonging to the *A. cajennense* species complex were identified according to Nava et al. [29] and Martins et al. [11].

Among the total 187 ticks collected from humans, 99 adult ticks (60 *A. cajennense* s.s., 20 *A. oblongoguttatum*, and 19 *A. coelebs*) were randomly selected for DNA extraction and were processed individually. DNA was extracted using the guanidine isothiocyanate and phenol/chloroform technique [30]. In order to validate the DNA extraction protocol, a conventional PCR (cPCR) assay targeting the mitochondrial 16S rDNA gene of ticks was performed, and one sample was negative. Therefore, DNA from 98 ticks was tested using a TaqMan real-time (qPCR) assay that targeted a 147-bp fragment of the rickettsial citrate synthase (*gltA*) gene. The qPCR-positive samples were subsequently tested using a conventional PCR (cPCR) assay using the primers Rr190.70p and Rr190.602n, which target the gene encoding the 190 kDa outer membrane protein (*ompA*) of SFG rickettsiae [15, 31–33]. A negative control (water) and a positive control (DNA of *Rickettsia rhipicephali*) were included in each PCR run.

The cPCR amplicons were purified using the Wizard^®^ SV Gel and PCR Clean-Up System (Promega, Madison, WI, USA), according to the manufacturer’s instructions. Sequencing was performed bidirectionally using the BigDye^™^ Terminator v3.1 Cycle Sequencing Kit (Applied Biosystems, Foster City, CA, USA) on an ABI 3500xL Genetic Analyzer, using the same primer set employed in the cPCR. The resulting sequences were compared against GenBank entries using the BLASTn algorithm (accessed 7 May 2025), to identify the most closely related *Rickettsia* species.

## Results

A total of 341 human serum samples were analyzed, of which 145 (42.5%) were seropositive for at least one of the five *Rickettsia* species. The ages of the seropositive individuals ranged from 16 to 45 years. Of these, 68 (47%) samples were collected in the municipality of Imperatriz, 45 (31%) from Açailândia, and 32 (22%) from São Luís. The number of reactive sera for each *Rickettsia* species was as follows: 135 (39.6%) for *R. rickettsii*, 136 (39.9%) for *R. bellii*, 142 (41.6%) for *R. amblyommatis*, 143 (41.9%) for *R. parkeri*, and 143 (41.9%) for *R. rhipicephali* (Table 1).

**Table 1:** Results of immunofluorescence assay for five *Rickettsia* species in humans from three locations in the State of Maranhão, Amazon biome, Brazil

Municipality	No. persons tested	No. person’s seroreative (%)	No. seroreactive persons to each of the *Rickettsia* species (% seroreactivity for the area)					No. persons with determined homologous reaction (PAIHR in parentheses)
			*Rickettsia rickettsii*	*Rickettsia parkeri*	*Rickettsia rhipicephali*	*Rickettsia amblyommatis*	*Rickettsia bellii*	
São Luís	136	32 (23.5)	30 (94%)	31 (98%)	32 (100%)	30 (94%)	31 (98%)	Indeterminate
Imperatriz	153	68 (44.4)	60 (88%)	67 (98%)	68 (100%)	68 (100%)	66 (96%)	2 (*R. bellii*); 1 (*R. rhipicephali*); 3 (*R. amblyommatis*)
Açailândia	52	45 (86.5)	45 (100%)	45 (100%)	43 (96%)	44 (98%)	39 (81%)	1 (*R. bellii*); 2 (*R. amblyommatis*)

PAIHR: possible antigen involved in a homologous reaction (serum showing for a *Rickettsia* species titer at least fourfold higher than that observed for any other *Rickettsia* species was considered homologous to the first *Rickettsia* species)

In São Luís, no human serum showed an endpoint titer to a *Rickettsia* species at least fourfold higher than the endpoint titers for the remaining *Rickettsia* species; therefore, no possible antigen involved in a homologous reaction (PAIHR) was determined in that municipality. Conversely, in Imperatriz, *R. rhipicephali* was considered the PAIHR in one individual, *R. amblyommatis* in three samples, and *R. bellii* in two. In Açailândia, *R. amblyommatis* was considered the PAIHR in two individuals, and *R. bellii* in a single sample (Table 1). Overall, IFA endpoint titers ranged from 64 to 1024 across the three municipalities, with possible homologous endpoint titers ranging from 512 to 1024 in Imperatriz, and from 256 to 1024 in Açailândia (Table 2).

**Table 2:** Endpoint titers of indirect immunofluorescence assay (IFA) for five *Rickettsia* species in humans in the State of Maranhão, Amazon biome, Brazil

Municipality	IFA titers					
	*Rickettsia rickettsii*	*Rickettsia parkeri*	*Rickettsia rhipicephali*	*Rickettsia amblyommatis*	*Rickettsia bellii*	PAIHR
Imperatriz	128	128	64	1024	128	*R. amblyommatis*
Imperatriz	64	64	1024	128	128	*R. rhipicephali*
Imperatriz	128	128	64	512	128	*R. amblyommatis*
Imperatriz	64	64	128	1024	128	*R. amblyommatis*
Imperatriz	64	64	64	64	512	*R. bellii*
Imperatriz	64	64	64	64	512	*R. bellii*
Açailândia	64	64	64	1024	64	*R. amblyommatis*
Açailândia	64	64	64	512	64	*R. amblyommatis*
Açailândia	64	64	64	64	256	*R. bellii*

PAIHR: possible antigen involved in a homologous reaction (serum showing for a *Rickettsia* species titer at least fourfold higher than that observed for any other *Rickettsia* species was considered homologous to the first *Rickettsia* species)

**Fig. 2:**
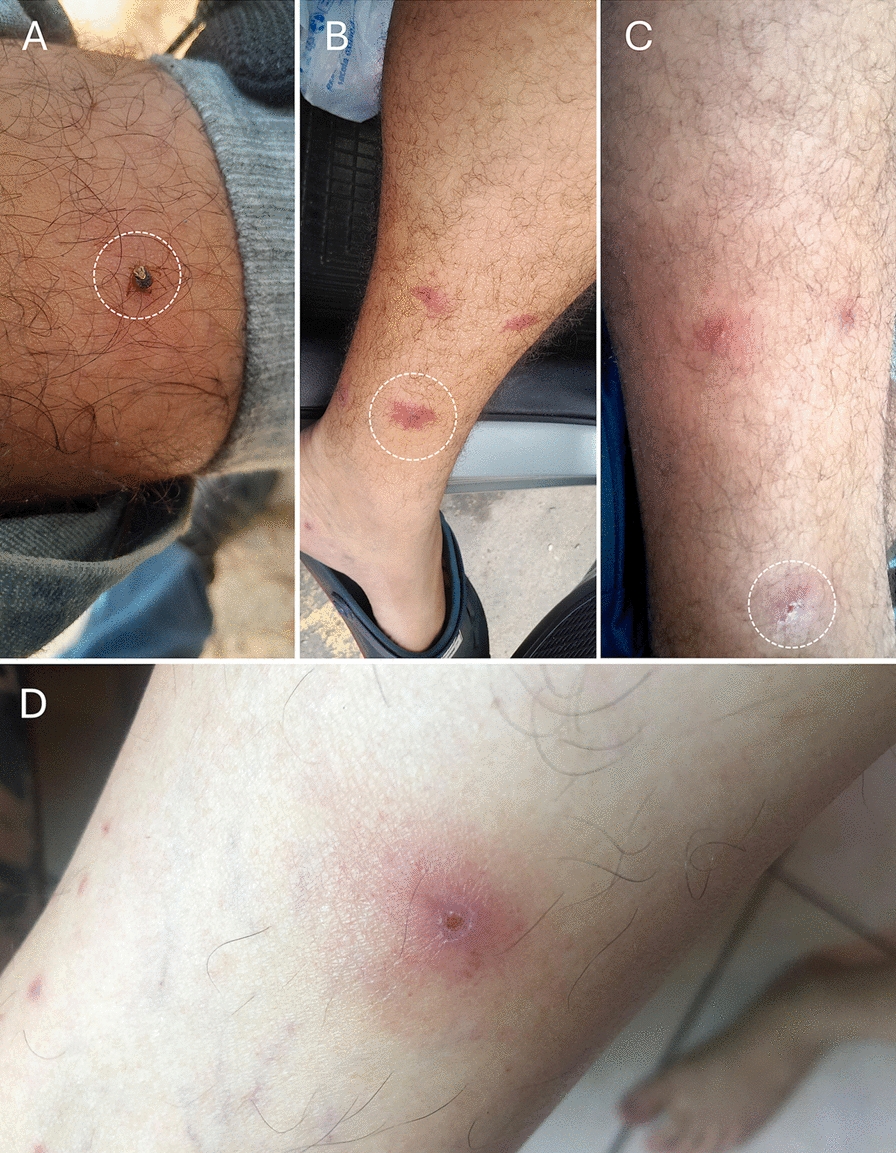
Lesions caused by *Amblyomma cajennense* sensu stricto. Individual 1 (**A**–**C**): (**A**—female of *A. cajennense* s.s. attached on the leg), (**B**—3 d after detachment) and (**C**—10 d after detachment). Individual 2 (**D**—10 d after detachment)

In total, 187 ticks (157 adults and 30 nymphs) representing five species were collected from 27 individual humans. An average of 6.9 ticks were collected per host, ranging from 1 to 12 (including both adults and nymphs). The most abundant tick species parasitizing humans was *A. cajennense* s.s., with 125 specimens (66.8%; 95 adults and 30 nymphs), followed by *A. oblongoguttatum* with 34 specimens (18.2%), *A. coelebs* with 24 specimens (12.8%), *A. sculptum* with 4 specimens (2.1%), and *Rhipicephalus sanguineus* sensu lato with 1 specimen (0.5%) (Fig. 2; Table 3). The greatest diversity of ticks on humans was recorded in Centro Novo do Maranhão (*A. oblongoguttatum*, *A. coelebs*, and *A. cajennense* s.s.), with emphasis on *A. oblongoguttatum* and *A. coelebs*, which were collected exclusively in this area. *A. cajennense* s.s. was the most common tick species parasitizing humans, and this association was recorded in six municipalities. No cases of co-infestation, with two or more tick species on the same individual host, were observed.

**Table 3:** Ticks collected on humans in seven municipalities of the State of Maranhão, eastern Amazon, Brazil

Tick species	No. parasitized humans	Total no. ticks (M.I.)^#^	No. ticks according to municipalities*						
			Aça	Imp	S.Inês	CNM	Ana	Bur	SLuis
*A. cajennense* s.s	11	125 (11.4)	12F	10F	13F, 8M, 30N	20F, 23M	2F, 4M	3F	
*A. oblongoguttatum*	8	34 (4.3)				21F, 12M		1F	
*A. coelebs*	4	24 (6.0)				14F, 10M			
*A. sculptum*	3	3 (1.0)	1F	2F					
*R. sanguineus* s.l	1	1 (1.0)							1F
Total	27	187 (6.9)	13	12	51	100	6	4	1

^#^M.I.: mean intensity of tick infestation (mean no. ticks/infested host)

*Aça, Açailândia; Imp, Imperatriz; CNM, Centro Novo do Maranhão; Ana, Anajatuba; Bur, Buriticupu; S.Luis, São Luís; F, females; M, males; N, nymphs

Lesions and dermatitis at the site of the tick bite were reported by many of the infested individuals with local dermatitis lasting between 3 and 5 d following removal of the tick. In some cases, it was possible to observe the progression of the lesion caused by the bite of *A. cajennense* s.s. (adult and nymph) (Fig. 2; individual 1 [A–C] and individual 2 [D]). These lesions were observed on the third day after tick removal and were characterized by an erythematous halo and intense pruritus. On the tenth day, a lesion resembling a small eschar was observed at the site of the tick bite (Fig. 2; individual 1 [A–C] and individual 2 [D]). The affected individuals reported fever, fatigue, and muscle pain within the first 24–48 h of parasitism.

A total of 99 (53%) tick specimens were screened by real-time PCR targeting the *gltA* gene of *Rickettsia* spp., with 33 (33.3%) samples testing positive. When stratified by tick species and collection site, *Rickettsia* DNA was detected in 4/9 (44.4%) *A. cajennense* s.s. adults from Açailândia, 22/42 (52.4%) from Centro Novo do Maranhão, and 0/8 (0.0%) from Imperatriz. Among *A. coelebs* adults from Centro Novo do Maranhão, 7/19 (36.8%) tested positive, while all *A. oblongoguttatum* adults from the same locality were negative 0/20 (0.0%) (Table 4). All 33 qPCR-positive samples for *gltA* gene of *Rickettsia* spp. were subjected to cPCR targeting the *ompA* gene of SFG *Rickettsia*. Of these, 32 yielded reliable amplicons. One *A. cajennense* s.s. specimen of the Centro Novo do Maranhão tested positive in the *gltA* qPCR but negative in the *ompA* cPCR.

**Table 4:** Molecular detection of rickettsial DNA in *Amblyomma* spp. adult ticks collected from humans in Maranhão State, northeastern Brazil, by cPCR targeting the 190-kDa outer membrane protein (*ompA*) gene

Tick species	Collection site*	No. ticks with rickettsial DNA/no. tested ticks (% positivity)	*Rickettsia* species identified by DNA sequencing (GenBank accession number)
*A. cajennense* s.s	Aça	4/9 (44.4)	No reliable sequence
	CNM	22/42 (52.4)	*R. amblyommatis* (KM262194)
	Imp	0/8 (0)	
*A. coelebs*	CNM	7/19 (36.8)	*R. amblyommatis* (MW147461)
*A. oblongoguttatum*	CNM	0/20 (0)	
Total		33/98 (33.7)	

*Aça, Açailândia; CNM, Centro Novo do Maranhão; Imp, Imperatriz

Reliable *ompA* sequences were obtained from six samples, three from *A. coelebs* and three from *A. cajennense* s.s. BLAST analysis showed that the *ompA* fragments (ranging from 381 to 475 bp excluding primer regions, and unreliable portions in the extremities of some sequences) were 100% identical to *R. amblyommatis* sequences available in GenBank. Specifically, the sequences from *A. cajennense* s.s. matched accession number KM262194, while those from *A. coelebs* matched accession number MW147461 (Table 4).

## Discussion

Studies on the seroprevalence of *Rickettsia* spp. in humans in Brazil have been concentrated in the southeast and south regions, especially in regions endemic for SF [34–38]. In the northeast region of Brazil, where the State of Maranhão is located, most SF cases have been reported in the Atlantic Forest biome, and were characterized as mild SF, the presence of an inoculation eschar, myalgia, and no deaths [5, 8, 23]. In two of these cases, *R. parkeri* was identified as the agent, and *A. ovale* was implicated as the vector. In the remaining cases (~15 cases), the agent was identified as *Rickettsia* sp., and the vector was not determined.

In Maranhão State, where the present study was conducted, four cases of SF have been officially confirmed according to federal reports [7], with two in the Cerrado biome and two in the Amazon biome. However, neither the *Rickettsia* species nor the vector was identified in any of these four cases. Curiously, no case of SF has been confirmed by the State Department of Zoonosis of Maranhão. This discrepancy indicates that there are many knowledge gaps concerning SF in this region, and studies on potential agents and vectors would be useful for understanding the disease and clarifying uncertain cases for both private and public health professionals. It is important to mention that some rickettsiae of the SFG (e.g., *R. amblyommatis*) circulate in both the Cerrado and Amazon biomes in Maranhão, potentially infecting ticks and humans in this region [12, 14]. Therefore, studies that investigate rickettsial agents in ticks and human exposure to these agents in the State of Maranhão are necessary and timely.

A total of 145 human samples were seropositive for *Rickettsia* spp. in the municipalities of Imperatriz (68 samples), Açailândia (45 samples), and São Luís (32 samples). The overall seroprevalence was 42.5%. This value was higher than those reported for other regions of Brazil, which have shown a seroprevalence of approximately 2–12% [34–38]. This difference may be related to peculiarities associated with the Amazon biome. The findings provide evidence that individuals from the municipalities of São Luís, Imperatriz, and Açailândia were exposed to *Rickettsia* spp. from the SFG, albeit without evidence for the development of clinical disease.

In Imperatriz and Açailândia municipalities, three species of *Rickettsia* were identified as the PAIHR: *R. bellii*, *R. rhipicephali*, and *R. amblyommatis*. Absence of PAIHR in sera from São Luís could be related to the lower seroprevalence in this municipality, when compared with the other two municipalities (Table 1). The bacterium *R. amblyommatis* was the most prevalent, being found in both municipalities, with possible homologous endpoint titers of 512 and 1024. A curious result was the titer of 1024 for *R. rhipicephali* in Imperatriz. To date, there are no reports of this agent in Maranhão, so caution should be exercised when interpreting this finding.

In Brazil, *R. bellii* has been reported to infect approximately 15 species of hard ticks, 8 of which were recorded parasitizing humans in the Amazon biome: *A. humerale*, *Amblyomma longirostre*, *A. oblongoguttatum*, *A. ovale*, *Amblyomma rotundatum*, *Amblyomma scalpturatum*, *A. tigrinum*, and *Amblyomma varium* [13, 15, 17, 22, 39–41]. This rickettsial agent is considered nonpathogenic to humans, although a large inoculum of this organism has been shown to cause local dermatitis in rabbits under experimental conditions [42].

*Rickettsia rhipicephali* has been detected in three species of hard ticks in Brazil: *Haemaphysalis juxtakochi*, *Amblyomma yucumense*, and *Amblyomma romarioi* (= haplotype Nazaré) [43–45]. Of these, only *H. juxtakochi* occurs in the Amazon and has been recorded parasitizing wild animals, domestic dogs, and humans [13, 27]. Serological studies have detected possible homologous titers to *R. rhipicephali* in domestic dogs in several regions of Brazil, including the Amazon biome [16, 19, 46]. Although still incipient, it is possible that the association between *H. juxtakochi* and domestic dogs may be contributing to the maintenance of *R. rhipicephali* in the Amazon biome. Although its pathogenicity remains undetermined, serological evidence from humans infected with *R. rhipicephali* in the USA has shown the presence of an inoculation eschar [47]. It is important to mention that cross-reaction between rickettsial agents of the SFG is very common [25, 48–50]. These cross-reactions can lead to misdiagnosis, especially in patients with nonspecific clinical manifestations. Therefore, our report of a single seropositive individual with *R. rhipicephali* as the PAIHR should be interpreted with caution.

Seroreactivity to *R. amblyommatis* was the most common result among humans in the present study. It was also the only agent infecting ticks collected from humans. This finding is corroborated by the wide circulation of this SFG agent in the Amazon biome, as evidenced by serological exposure to *R. amblyommatis* in domestic animals [12, 19, 46, 51] and by its direct detection in ticks [12, 14, 15, 17, 22]. *Rickettsia amblyommatis* has been recorded infecting a variety of ticks in Brazil, of which six species stand out for parasitizing humans in the Amazon biome: *Amblyomma longirostre*, *A. cajennense* s.s., *A. oblongoguttatum*, *A. coelebs*, *A. sculptum*, and *Amblyomma varium* [12–14, 22, 27]. All these tick species have established populations in Maranhão State, including in the Amazon biome [12–14].

A total of 187 ticks were collected while parasitizing humans in our study. The average of 6.9 ticks per infested human is similar to that observed by Szabó et al. [52] (5.7 ticks per infested human) in the Cerrado biome, and below the records of Ramos et al. [53] (12.7 ticks per human) in the Pantanal biome. Some tick specimens (e.g., larvae and nymphs) were carried by people but were not collected, indicating that the average infestation rate on humans (6.9) may be even higher in the Amazon biome. The species *A. cajennense* s.s. and *A. oblongoguttatum* were the most commonly encountered parasitizing humans in our study, a phenomenon previously reported by Luz et al. [13]. Among these ticks, 26 specimens of *A. cajennense* s.s. and 7 *A. coelebs* were positive for *R. amblyommatis*, mainly from humans in Centro Novo do Maranhão. Our findings are consistent with previous studies reporting high infection rates of *R. amblyommatis* in *Amblyomma* spp. across Brazil and Central America, including molecular detection and successful isolation from *A. mixtum* in Panama [54], *A. americanum* in the USA [22], and *A. cajennense* s.s. and *A. coelebs* in the Amazon biome [14, 22].

In common with the other agents to which PAIHR was detected in the present study, the pathogenicity of *R. amblyommatis* is undetermined. Yet, several studies suggest that this agent may cause disease in mammals, presenting as a mild and self-limiting febrile condition, and may also elicit cross-protective immunity against more virulent SFG *Rickettsia* species [22, 55–57]. In the USA, in regions with human cases of SF, the absence of *R. rickettsii* has led to speculation that *R. amblyommatis* may be the etiological agent [22]. In this context, Billeter et al. [58] reported infection by *R. amblyommatis* in a tick biting a man who developed a rash at the site of the bite, and the same authors also reported that soldiers from Georgia (USA) developed a fever after contact with *A. americanum* and were seropositive for *R. amblyommatis*. In addition, ticks collected from these patients tested positive for *R. amblyommatis* through molecular analysis. In North Carolina (USA), Apperson et al. [58] collected ticks in an area with human cases of SF, and after molecular analysis, determined that approximately 45% were infected with *R. amblyommatis*. Although there is some serological evidence that *R. amblyommatis* can cause SF, to date, this agent has not been isolated from human patients.

Lesions and dermatitis were observed in many individuals parasitized by *A. cajennense* s.s. nymphs and adult ticks in the current study (Fig. 2). In fact, this tick species is highly anthropophilic and aggressive toward humans, and is one of the most common in the Amazon biome, confirming reports in the literature that ticks of the *Amblyomma cajennense* complex are the most aggressive ticks that bite humans in the Neotropical region [22, 27, 52, 59–61]. In some cases, it was possible to monitor the evolution of the lesion (Fig. 2), where on approximately the 8th to the 12th day after tick removal, an eschar appeared at the bite site. In general, severe lesions that evolve into an eschar occur due to pharmacological components of tick saliva in combination with host sensitivity resulting from previous exposure [61]. This type of lesion (known as toxicosis) is most often caused by Argasidae ticks, as seen with *Ornithodoros brasiliensis* and *Ornithodoros mimon* [62–64], including cases of human bites by *Ornithodoros rietcorreai* in Maranhão State [62].

Another hypothesis is the presence of a pathogen in the tick’s saliva, e.g., *Rickettsia* sp., a factor not investigated in the present study. However, both individuals who developed eschars were infested in a degraded area of eastern Amazonia in the municipality of Santa Inês, where de Araújo et al. [14] reported a high population density of *A. cajennense* s.s. in the environment, and approximately 7% of these ticks were positive for *R. amblyommatis*.

## Conclusions

This is the first serological study for the detection of anti-*Rickettsia* antibodies in humans and the detection of *R. amblyommatis* in ticks parasitizing humans in the Amazon biome. The detection of *R. amblyommatis* in human-biting ticks, along with concurrent seropositivity in human sera from the same region, supports the hypothesis that this agent is actively circulating in the Amazon biome and may be responsible for undiagnosed cases of nonlethal spotted fever in Maranhão State. *Amblyomma cajennense* s.s. showed a high rate of infection by *R. amblyommatis* and was also the most common tick parasitizing humans in the study area. This suggests that *A. cajennense* s.s. is a strong candidate as a biological vector of this agent for animals and humans in the Amazon biome. However, we cannot rule out possible transmission by *A. coelebs*.

These findings highlight the need to include *R. amblyommatis* in the differential diagnosis of febrile illnesses in the region and underscore the importance of enhanced tick surveillance to better understand its epidemiological role. All agents detected in humans in the present study have undetermined pathogenicity. However, it should be emphasized that many *Rickettsia* species now recognized as pathogenic to humans were initially considered nonpathogenic or of unknown pathogenicity. Therefore, the potential role of *R. amblyommatis*, *R. rhipicephali*, and *R. bellii* as human pathogens in the Amazon biome cannot be discounted.

## Data Availability

Data supporting the main conclusions of this study are included in the manuscript.
